# High Thermal Stability and Low Dielectric Constant of BCB Modified Silicone Resins

**DOI:** 10.3390/polym15132843

**Published:** 2023-06-28

**Authors:** Hubo Wei, Xian Li, Xu Ye, Chao Guo, Juan Peng, Jiaying Liu, Xinyu Hu, Junxiao Yang, Jinxiang Chen

**Affiliations:** 1 School of Materials and Chemistry, Southwest University of Science and Technology, Mianyang 621010, China; 15228732958@163.com (H.W.); guochao07262022@163.com (C.G.); yangjunxiao@swust.edu.cn (J.Y.); 2 State Key Laboratory of Environmentally-Friendly Energy Materials, Southwest University of Science and Technology, Mianyang 621010, China; 3 School of Continuing Education, Southwest University of Science and Technology, Mianyang 621010, China

**Keywords:** silicone resin, benzocyclobutene, dielectric constant, thermal stability, hydrolytic condensation

## Abstract

Based on the excellent physical properties and flexible molecular modifiability, modified silicone resins have received favorable attention in the field of microelectronics, and recently a number of modified silicone resins have appeared while few breakthroughs have been made in low dielectric constant (low-k) materials field due to the limitations of structure or the curing process. In this work, functional silicone resin with different BCB contents was prepared with two monomers. The resins showed low dielectric constant (*k* = 2.77 at 10 MHz) and thermal stability (T_5%_ = 495.0 °C) after curing. Significant performance changes were observed with the increase in BCB structural units, and the functional silicone obtained does not require melting and dissolution during processing because of good fluidity at room temperature. Moreover, the mechanical properties of silicone resins can be also controlled by adjusting the BCB content. The obtained silicone resins could be potentially used in the field of electronic packaging materials.

## 1. Introduction

Silicone resin is an organicinorganic hybrid material with SiOSi chains and has been widely used in the fields of microelectronic [1,2,3], aerospace [4,5], and nuclear industries [6] due to its high thermal stability, low dielectric constant (low-k), and good mechanical properties. Silicone resin has great adjustability that could easily enhance or change its properties by adjusting organic groups. Usually, a phenyl group could be used to improve the thermal stability [7], fluorine [8] and ureido radical [9] could be used to enhance the tracking resistance, and noble metal [10,11,12] could also be used to increase the photoelectric and magnetic effect. Due to this, silicone resin has been widely studied as low-k [13,14] and high-k [15,16] material in recent years. Low-k material is mainly used as the interlayer dielectric of electronic devices and the substrate of circuit boards. The progress of low-k materials has benefited from the development of communication technology and the microelectronics field. According to the signal transmission speed formula:

$$V=\frac{k\cdot c}{\sqrt{\xi }}$$

where *k* and *c* are constants and *ξ* is the dielectric constant of the material. It shows that the signal transmission speed is negatively correlated with the dielectric constant of the material. Therefore, for the field of low-dielectric materials, how to reduce the dielectric constant is the focus of further research.

In order to reduce the dielectric constant of silicone resin, it is necessary to reduce the dipole moment and the number of dipoles, usually by introducing non-polar groups such as phenyl (*k* = 3.31–3.37 at 7–17 GHz) [17] and large-volume structure such as hollow glass microspheres (*k* = 2.40 at 5 to 30 MHz) [18]. Compared with the conventional vinyl silicone resin, the modified silicone resin has obvious advantages of low-k and thermal stability. The curing methods of most silicone resins mainly include hydrosilylation [19,20,21] and epoxy cross-linking reaction [22,23]. The silicone resin with epoxy groups has excellent optical properties after curing, but due to the existence of C–O bonds, the thermal stability (T_5%_ > 200 °C) and low-dielectric properties (*k* = 2.9–3.6 at 10 MHz) [3] of epoxy cross-linked silicone resin are often insufficient, and the hydrosilylation reaction needs to be cured by adding catalysts such as chloroplatinic [24] and molydbenumsilyl [25], which can influence the dielectric properties of silicone resins. With the development of microelectronics technology, the performances of silicone resins have higher requirements.

BCB is a kind of benzene compound with openable side chains and can be cross-linked by Diels-Alder reaction above 200 °C with no catalyzer and no small molecules are generated. The silicone resins with more BCB groups have better performance compared with those with phenyl groups. BCB has good application prospects in many fields, such as bonding materials [26], liquid crystals [27], sensors [28,29], and microelectronics [30,31]. Compared with other functional silicones, BCB functionalized silicone resin has a lower dipole moment, which can effectively reduce the dielectric constant, and the benzene ring structure involved in the construction of the cross-linked network will bring additional strength.

In recent years, the BCB modified silicone resins have been widely researched in low-k materials [32,33], and many of them show low-k (2.27~2.70) [34,35] and good thermal stability (T_5%_ > 450 °C) [36]. Cheng et al. [37] synthesized BCB chlorosilane via Grignard reaction and designed and prepared a series of BCB modified siloxanes with low-k (2.65~2.78) and high thermal stability (T_5%_ > 440 °C). The synthesis of most BCB silicone monomers requires strict reaction conditions and often requires multiple synthesis steps to obtain target monomers with a large molecular structure. It is also reported that BCB has been introduced into the long chain of siloxane [38,39], and the preparation conditions of BCB modified linear siloxane obtained by hydrolysis condensation are relatively simple, due to the high molecular weight of linear silicones, and its performance was better. It is interesting that the structures of BCB modified siloxanes reported in the literature were slightly different, which also leads to some differences in their properties, especially in the *k*.

At present, the melting point and glass transition temperature of BCB siloxane products are too high, which requires solvent or high temperature treatment in the subsequent processing process, resulting in a large amount of energy and resource consumption. More detailed experiments are needed on how to explore the relationship between the properties and environmentally friendly applications. In this work, two siloxane monomers, benzocyclobutenyldimethylethoxysilane (BDMES) and benzocyclobutenylmethyldiethoxysilane (BMDES), were synthesized via Grignard reaction. Subsequently, a simple mild method for the production of BCB functional polymer was designed with hydrolytic condensation of two monomers with dimethyldiethoxysilane (DMDES). The mechanical properties and thermal stability of the cured resins increased obviously with the increasing BCB content, and the dielectric constant and the coefficient of thermal expansion (CTE) of the functional silicones had a regular decline. This work could inspire the design and performance studies of modified silicone resins.

## 2. Materials and Methods

### 2.1. Materials

4–Bromobenzocyclobutene (4–Br–BCB, 98%) was purchased from Beichuan Tiannuo Photoelectric Material Co. Ltd. (Mianyang, China). Diethoxydimethylsilane (DEDMS, 95%) and methyltriethoxysilane (MTES, 95%) were purchased from China Bluestar Chengrand Co. Ltd. (Chengdu, China). Ethanol (C_2_H_5_OH), tetramethylammonium hydroxide (TMAOH), toluene, magnesium (Mg), and iodine (I_2_) were purchased from Chengdu Chron Chemical Co. Ltd. (Chengdu, China). Tetrahydrofuran (THF) was purchased from J&K Scientific (Shanghai, China) and purified before use.

### 2.2. Experiment

#### 2.2.1. Synthesis of Benzocyclobutenyldimethylethoxylsilane (BDMES)

**Scheme 1:**
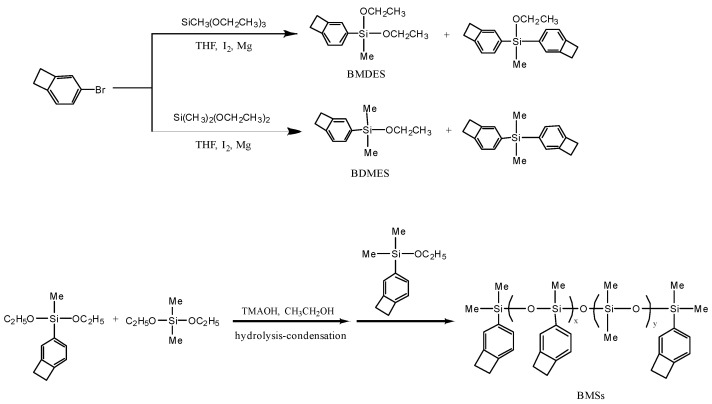
Route of BCB modified silicone resins.

Using magnesium ribbons (2.64 g, 110 mmol) and iodine pellets (0.05 g, 0.2 mmol) as initiators, under a nitrogen atmosphere, 30 mL of purified THF was injected and 4–Br–BCB (18.30 g, 100 mmol) dissolved in 30 mL of THF was added dropwise at room temperature with continuous stirring. The reaction mixture was maintained at 50 °C for 2 h and continuously stirred before naturally cooling to room temperature, then cooled down with ice water. The supernatant liquid was extracted as a Grignard reagent for the next step. In a nitrogen atmosphere, dimethyldiethoxysilane (15.80 g, 100 mmol) with 15 mL of purified THF was stirred for 2 h under −70 °C, then the Grignard reagent was added dropwise and reacted for 5 h. The chemical pathways were given in Scheme 1 below.

After the reaction system returned to room temperature, the reaction mixture was quickly poured into petroleum ether and the precipitation was filtered away. The liquid was collected and evaporated, and BDMES was obtained by vacuum distillation as colorless liquid (13.8 g, yield: 77.5%). FTIR: 3056, 2966, 1433, 1395, 1100, 1080, 820, 782 cm^−1^. ^1^H NMR (600 MHz, CDCl_3_, ppm): 0.27 (s, 3H, Si–CH_3_), 1.09 (t, 3H, O–C–CH_3_), 3.10 (d, 2H, Ar–CH_2_–C), 3.58 (dd, 2H, O–CH_2_–C), 6.98 (d, 1H, ArH), 7.18 (s, 1H, ArH), 7.34 (d, 1H, ArH).

#### 2.2.2. Synthesis of Benzocyclobutenylmethyldiethoxylsilane (BMDES)

The synthesis method of BMDES was similar to that of BDMES, using methyltriethoxysilane (20.8 g, 109.5 mmol) as raw material, and BMDES was obtained as colorless liquid (14.9 g, yield: 65.0%). FTIR: 3056, 2966, 1433, 1395, 1100, 1080, 819, 781 cm^−1^. ^1^H NMR (600 MHz, CDCl_3_, ppm): 0.20 (s, 3H, Si–CH_3_), 1.10 (t, 3H, O–C–CH_3_), 3.05 (d, 2H, Ar–CH_2_–C), 3.66 (dd, 2H, O–CH_2_–C), 6.95 (d, 1H, ArH), 7.19 (s, 1H, ArH), 7.35 (d, 1H, ArH).

#### 2.2.3. Preparation of BCB Modified Methyl Silicone Resins (BMSs)

First, 50 mL of ethanol, 0.02 g of TMAOH, and 5 mL of H_2_O were mixed homogeneously, and the mixture of BMDES (6.18 g, 30 mmol) and DMDES (4.44 g, 30 mmol) was added dropwise with stirring at room temperature for 4 h. Then, adequate BDMES was added and the reaction mixture was kept at 60 °C for 4 h. After the reaction, with toluene extraction, ultrapure water washing, anhydrous sodium sulfate drying, and precipitation filtration, the toluene solution was made by vacuum distillation. After vacuum drying for 12 h at 60 °C, the BMS was obtained as colorless transparent viscous liquid (yield of about 90%). The BMSs with different BCB contents were obtained by adjusting the proportion of BMDES and DMDES.

### 2.3. Preparation of Cured Silicone Resins

The cured BCB modified silicone resins were obtained in glass tubes under vacuum conditions. The heating program of the curing box was set at 120 °C, 160 °C, 180 °C, 200 °C, 220 °C, and 240 °C, and each temperature segment was maintained for 2 h. Cooling to about 60 °C naturally, the cured resins were obtained and polished as circular cylinders.

### 2.4. Characterization

Fourier transform infrared (FTIR) spectra from 4000~400 cm^−1^ were obtained by a Fourier transform infrared spectrometer (Nicolet iS50, Thermo Fisher, Boston, MA, USA) at 25 °C, and the simple pieces were made by spreading on KBr plate. ^1^H NMR spectra were obtained with a superconducting nuclear magnetic resonance spectrometer (AVANCE Ⅲ 600 MHz, Bruker, Karlsruhe, Germany) at 600 MHz with deuterated chloroform as solvent. The viscosities of resins at 25 °C were obtained by a revolving viscometer (C-PTD 180/AIR/QC, Anton Paar, Graz, Austria) at 800 rpm, and the complex viscosities of resins were measured by an HR-10 rotational rheometer in oscillating mode (1 Hz) at the heating rate of 3 °C min^−1^. Thermogravimetric analysis (TGA) and differential scanning calorimetry (DSC) (SDTQ600, New Castle, TA, USA)were used with an Al_2_O_3_ crucible (open) and performed on a TGA/DSC synchronous thermal analyzer (Mettler Toledo, Zurich, Switzerland) in nitrogen atmosphere at the heating rate of 10 °C min^−1^. The thermal expansion coefficients were determined using a static mechanical thermal analyzer (TMA 450, New Castle, TA, USA) with the heating rate of 5 °C/min from 30 to 300 °C. The *k* and dielectric loss of the resins were measured by an impedance analyzer (Agilent 4294A, Santa Clara, CA, USA) at room temperature, the frequency of the applied oscillation electric field was from 100 KHz to 20 MHz, the contact material was copper, AC voltage amplitude was 5 V, the samples were cylinders with a radius of 3.05 mm, the thicknesses of samples were according to measurement results, and the flat surfaces of the samples were gold-plated by sputter-coating. The hardness, elastic modulus, and loadingunloading characteristic curves were obtained from nanoindentation (G200, Agilent, CA, USA), with the surface approach velocity of 10 nm s^−1^ and 2000 nm depth.

## 3. Results

### 3.1. Synthesis of BDMES and BMDES

The BCB modified monomers and resins were prepared as shown in Scheme 1. BMDES and BDMES were synthesized via Grignard reaction and distilled with decompression from 70~90 °C and 80~110 °C, respectively. The ultimate products were transparent colorless liquids. In this work, Grignard reaction was highly targeted with few by-products, and the anaerobic non-water condition and low temperature suppressed the formation of dimer and polymer. In order to avoid the hydrolysis of monomers in the process of silica gel chromatographic separation, the difference in boiling points of various substances in the reaction was used for decompression separation, and the yields of BDMES and BMDES were 77.5% and 65.0%, respectively.

**Figure 1:**
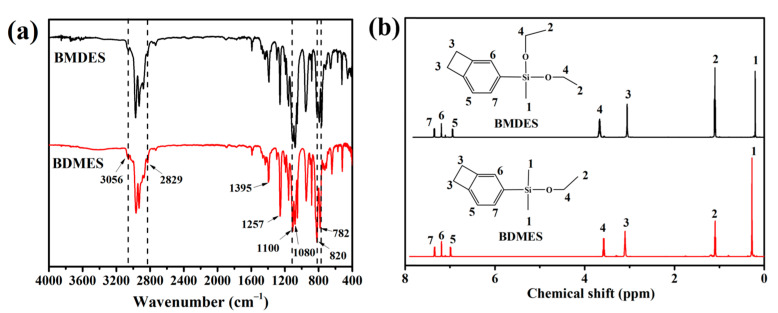
(**a**) The FTIR spectra of BDMES and BMDES. (**b**) The ^1^H NMR spectra of BMDES and BDMES.

The FTIR spectra of BMDES and BDMES are shown in Figure 1a. In the FTIR spectrum of BMDES, the absorption peaks at 3056, 1590, 1462, 820, 782 cm^−1^ showed the composition of 1, 2, 4-substituted benzene. The absorption peaks at 2966 cm^−1^ and 2930 cm^−1^ were assigned to the telescopic vibration of C–H on alkoxy. The absorption peak at 2829 cm^−1^ was thought to be a four-membered ring at phenyl. The absorption peaks at 1395 and 1257 cm^−1^ showed the existence of Si–CH_3_. The absorption peaks at 1100 cm^−1^ and 1080 cm^−1^ were assigned to the C–O and Si–O telescopic vibration, respectively. In the FTIR spectrum of BDMES, the BCB structure and the bond of C–H and Si–O were also found at the same absorption location as in BMDES, because of the similar structure. BMDES and BDMES contained the same group species, so the same resonance absorption was observed in two curves at various wavenumbers in the IR spectroscopy.

The ^1^H NMR spectra of BMDES and BDMES are shown in Figure 1b. In the ^1^H NMR spectrum of BMDES, the methyl peak of silicone appeared at 0.20 ppm. The ^1^H NMR signal assignment of methyl and methylene in –Si–O–CH_2_–CH_3_ appeared at 1.10 ppm and 3.66 ppm. The hydrogen absorption peaks at 3.10 ppm are consistent with the theoretical location of the cyclobutene. The resonance peaks from 6.95 ppm to 7.35 ppm were assigned to the three hydrogen atoms on the aromatic ring because of the different chemical positions. The resonance peak of BDMES was in the same position as that of BMDES, so the ^1^H NMR spectrum of BDMES was not analyzed separately. The chemical shift of resonance peaks in the spectrum was consistent with the theoretical chemical shift of BMDES and BDMES, and the ratio of the integral area was consistent with the number of H atoms in the molecular formulas. It manifested that BMDES and BDMES were synthesized successfully.

### 3.2. Preparation of BMSs

In this work, the content of BCB functional groups in BMS was adjusted by controlling the ratio of raw materials, and the raw material ratio and final yield of BMS are shown in Table 1. The content of BCB in BMS was calculated by the integral area of different H chemical shifts in the ^1^H NMR spectra. Due to more BDMES being added, the space hindrance and activity of silicone chain declined, which caused the yield to drop slightly.

**Figure 2:**
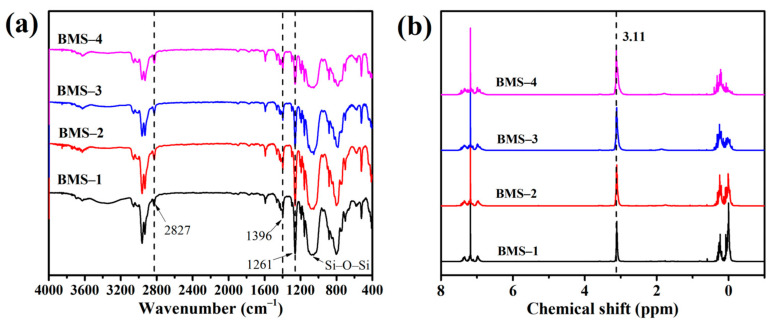
(**a**) The FTIR spectra of BMSs. (**b**) The ^1^H NMR spectra of BMSs.

Figure 2 shows the FTIR and ^1^H NMR spectra of the series of BMSs. According to the FTIR spectra of BMSs, instead of the flexural vibration absorption peak of Si–O in Si–O–CH_2_–CH_3_ at 1080 cm^−1^, the broad peaks of stretching vibration of Si–O–S appeared at 1100 cm^−1^ to 1000 cm^−1^, which indicated that the hydrolytic condensation reaction of siloxane occurred to form Si–O–Si chains. The bending vibration characteristic peaks of C–H bonds in silicone were observed at 1396 cm^−1^ and 1261 cm^−1^. The peaks from 1600 cm^−1^ to 1400 cm^−1^ were considered as the skeleton vibration of benzene rings in BMSs, and the peak at 2820 cm^−1^ was associated with methylene of cyclobutane in BCB. In ^1^H NMR spectra, the peaks at 1.1 ppm and 3.66 ppm are considered to be ethoxy and disappeared after hydrolytic condensation relative to Figure 1b. The proton absorption peaks from 5.89 ppm to 7.40 ppm were assigned as the H on aromatic nucleus structures. The peak at 3.11 ppm was thought to be methylene of cyclobutane in BCB. The main two peaks from 0.2 ppm to 0 ppm were considered to be resonance absorption of Si–CH_3_ belonging to BMDES and DMDES, respectively.

### 3.3. Viscosity of BMSs

**Figure 3:**
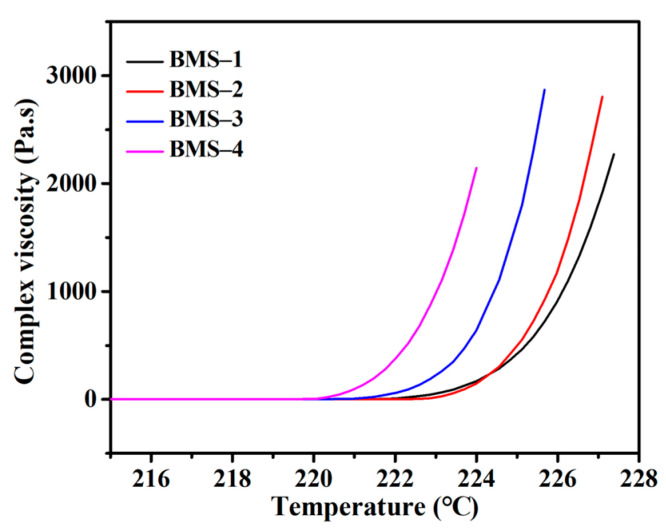
The complex viscosity curves of BMSs.

As shown in Table 2, the viscosity of BMS varied from 43.5 mPa s to over 2000 mPa s with changes in the raw material ratio. With the increasing BCB modified structural units in BMSs, the viscosity of BMSs changed more significantly, which showed that the viscosity of BMSs under normal conditions was obviously affected by the content of BCB. According to the complex viscosity curves obtained utilizing a rheometer (Figure 3), it can be seen that the complex viscosities of BMSs varied with the curing temperature. The cross-linking curing behavior of the silicone resins was investigated by characterizing the change in complex viscosity in the process of temperature change. The increase in complex viscosity was also correlated with the content of BCB, and significant large-scale intermolecular entanglement occurred 3 °C earlier in BMS-4 than in BMS-1. It indicated that the cross-linking conditions of silicone resins containing BCB were affected by the raw materials. Interestingly, an advanced increase in viscosity was obtained in BMS-1 with lower BCB content and, as the curing reaction went on, the viscosity increase in BMS-1 slowed down compared with those of others. It indicated that the initial low cross-linking unit density could block the free movement of the chains.

### 3.4. Thermal Stability of Cured BMSs

**Figure 4:**
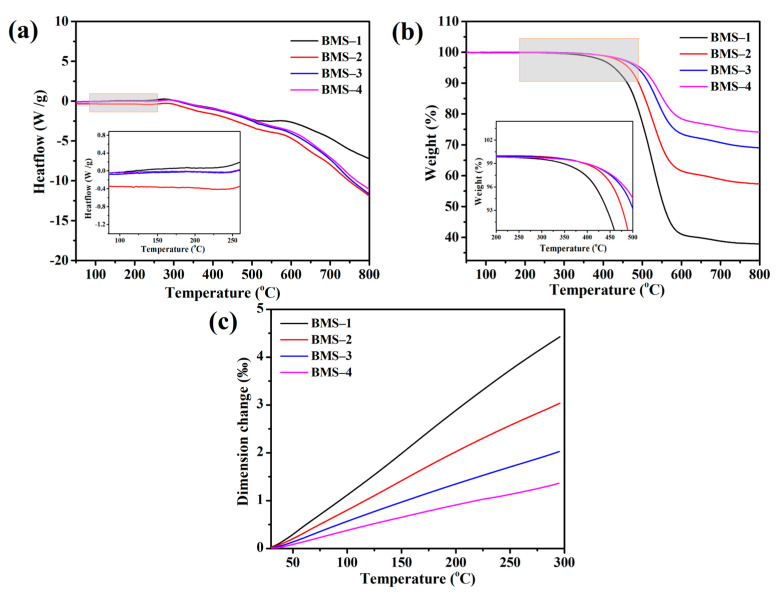
(**a**) The DSC curves of cured BMSs. (**b**) The TGA curves of cured BMSs. (**c**) The thermal expansion curves of cured BMSs.

As shown in Figure 4a, there was no prominent endothermic peak for BMSs under 250 °C, therefore the BCB was cross-linked completely. According to Figure 4b, with the BCB content increasing from 26.28 to 81.76%, the T_5%_ values of BMSs were 427.8, 464.8, 486.3, and 495.0 °C, and the char yields were 37.9%, 57.3%, 69.01%, and 74.0%, respectively. The thermal stability of the resins was enhanced with the increasing proportion of BCB groups. The char yield showed great change as an intramolecular rearrangement of siloxane happened when phenyl groups existed in Si–O–Si chains and, with the increase in Si–Ph structure in silicone resin, the T_5%_ and the residue at 900 °C grew significantly [40]. So, if there were alkyl groups such as phenyl in the Si–O–Si chain of silicone, its structure would be rearranged to obtain a more stable silicone heterocyclic structure at high temperature, which would make the char yield have such a change. After curing, the cross-linked network of BMS was formed by BCB units and Si–O chains, which caused the polydispersity to change and the char yield of BMSs increased at a high temperature comparing with other BCB silicone resins [41,42]. It was noted that there was no linear increase, the T_5%_ and char yield were elevated with the increasing BCB content in the resins, and the increasing range of the thermal stability due to BCB groups in the Si–O–Si structure was limited.

The thermal expansion properties of BMSs are shown in Figure 4c. The average thermal expansion coefficients of the cured BMSs were 164.8, 112.9, 75.97, and 51.57 ppm °C^−1^, respectively. Similar to the regularity of thermal stability, the TMA curves showed that the coefficients of the rate of the cured BMSs’ thermal expansion were slowly reduced from BMS-1 to BMS-4. The thermal expansion coefficient of materials remained constant during the testing process, and that of BMS-4 could reach the level of DVS-BCB (47 ± 3 ppm °C^−1^) [43,44]. This indicates that the thermal expansion properties of BMSs are closely related to the cross-linked density, the combination of BCB groups and Si–O–Si chains in BMS-1 made the cured BMS have a relatively high-density cross-linked network, and the effect of adding more BCB groups on the thermal expansion coefficient of BMSs gradually decreased.

### 3.5. Dielectric Properties of Cured BMSs

According to the Debye equation, the real and imaginary components of the complex dielectric coefficient of the material in the assigned frequency were calculated:

$$\xi \prime (\omega )=\xi_{\infty }+(\xi_{s}-\xi_{\infty })\frac{1}{1+\omega^{2}\tau^{2}}$$

$$\xi \prime \prime (\omega )=(\xi_{s}-\xi_{\infty })\frac{\omega \tau }{1+\omega^{2}\tau^{2}}$$

The dielectric loss tangent (tan*σ*) is the ratio of active and reactive vector components, according to the above formula, and the formula of dielectric loss tangent is as follows:

$$\tan\sigma =\frac{\xi \prime \prime }{\xi \prime }=\frac{(\xi_{s}-\xi_{\infty })\omega \tau }{\xi_{s}+\xi_{\infty }\omega^{2}\tau^{2}}$$

*ξ*_∞_ and *ξ_s_* are the optical frequency dielectric coefficient and the static dielectric coefficients, respectively, *ω* is the frequency of applied electric field, *τ* is the time constant of relaxation polarization. The capacitance of series (*C*_p_) and tan*σ* of the resins were obtained through data acquisition and calculation by the instrument program.

**Figure 5:**
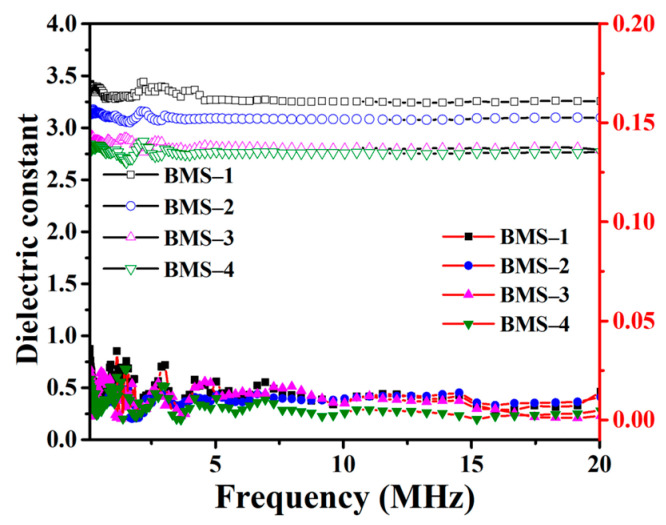
The dielectric constant and dielectric loss curves of BMSs.

The *k* of the resin was calculated as the following equation:

$$k=\frac{C\cdot d}{\xi_{0}\cdot S}$$

where *C*, *d*, and *S* represent the capacitance, thickness, and cross-sectional area (CSA) of the BMS sample, respectively. *ξ*_0_ represents the vacuum dielectric constant of 8.854 × 10^−12^ F m^−1^. Figure 5 shows the curves of the *k* and dielectric loss of BMSs with the frequency range from 100 KHz to 20 MHz. Below the frequency of 20 MHz, the *k* values of BMSs did not change significantly, and they were 3.30, 3.10, 2.84, and 2.77 at 10 MHz, respectively. The dielectric loss curves of BMSs show irregular changes but little fluctuation, and the tan*σ* values of BMSs were less than 1% at 10 MHz. The tan*σ* of BMS-4 is 3.59 × 10^−3^ and the dielectric loss is lower than that of DVS-BCB (tan*σ* = 8 × 10^−3^ at 1 MHz), which is widely used for low-k material.

The *k* is related to the number of dipoles in unit volume. With the increasing content of BCB, more benzene ring structures replaced Si–O–Si bonds with stronger polarity, which resulted in decreasing the *k*. The results showed that there was a boundary effect on reducing the *k* of BMSs by decreasing the content of BMDES, but the presence of Si–O–Si chains inhibited the further reduction of the *k*. According to Table 3, the dielectric property of BMS is slightly better than that of cured BCB functionalized silicone monomers, and it is similar to certain BCB silicone resins with similar long chains. The free volume of silicone resin can be increased by obtaining molecules with larger molecular weight and volume before curing.

### 3.6. Mechanical Properties of Cured BMSs

**Figure 6:**
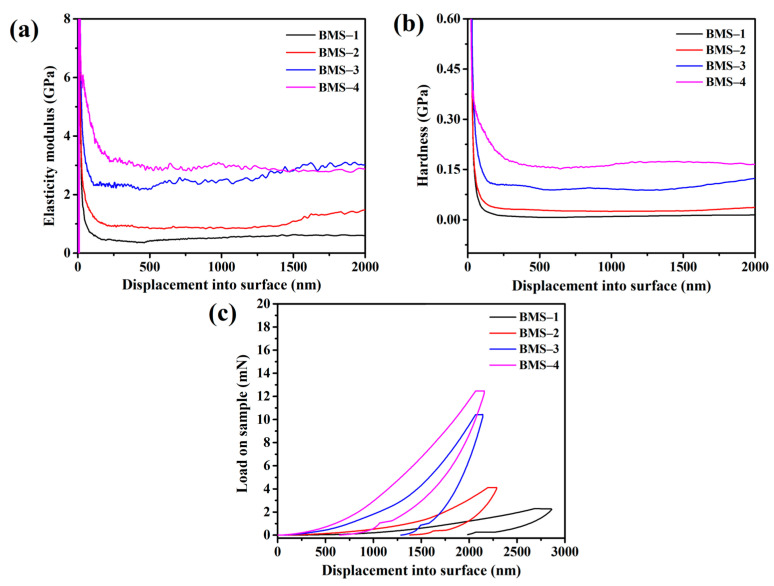
(**a**) The hardness curves of cured BMSs. (**b**) The BMSs’ variation curves of elastic modulus with indentation depth. (**c**) Loadingunloading curves against displacement into surface of cured BMSs.

The mechanical properties of BMSs were determined by nanoindentation testing at a press speed of 10 nm s^−1^. The hardness, elastic modulus, and loadingunloading curves against displacement into the surface are shown in Figure 6. The average hardnesses of cured BMSs were 0.02, 0.05, 0.12, and 0.24 GPa, respectively. The hardness of BMSs increased with the increasing BCB content, as it profited from the rigid groups of BCB structures in the resins. Moreover, the elastic modulus increased from 0.5 GPa to 3.7 GPa. According to Figure 6b, it appeared that the elastic modulus of BMS-3 and BMS-4 had slight differences compared with those of others, which could be because the movement of the molecular chain in the resins was restricted by the cross-linking network when the content of BCB in the resin increased to a certain extent. This can be reflected by the storage modulus of the materials which exhibited similar properties to the loadingunloading curves in another report [45].

## 4. Conclusions

With the continuous exploration of siloxane resins in dielectric fields, BCB functional silicone resins show excellent performance. It is necessary to carry out further research on the conditions which affect the properties of BCB silicone resins. In this work, two kinds of BCB modified siloxane monomer with different quantities of ethoxy were successfully synthesized and a series of resins with different BCB contents were obtained via hydrolysis condensation reaction. The viscosity, thermal stability, dielectric properties, and mechanical properties were investigated. The melting points of the synthesized BCB functionalized silicone resins were so low that they maintained good fluidity at room temperature. The proportion of BCB groups could make the dielectric and mechanical properties of cured resins qualitatively increase, the *k* decreased from 3.30 to 2.77, the hardness increased from 0.02 to 0.24 GPa, and the elastic modulus increased from 0.5 to 3.7 GPa. The viscosity and curing process of the resins were affected obviously by the proportion of BCB groups and, with the increasing BCB content, the amount of rigid groups and the cross-linking degree of chains in BMSs can be improved at the same time, which endowed the cured BCB functional silicone resins with high thermal stability and low-k. The changes in those properties conformed to a certain rule and the properties and processability of the materials could be balanced by adjusting the content of BMDES in the resins flexibly. It is worth noting that BMS processing before curing does not require high temperature or dissolution by solvent, and this not only benefits the direct use of BMS as a low-dielectric material, but also facilitates its composition with other materials. In addition, the module-like functionalization of silicones can also be used to obtain silicones of other structures.

## Figures and Tables

**Table 1:** The comparisons of different functional silicones with low dielectric constant.

Resins	Dielectric Constant (1 MHz)	Ref.
DVS-BCB	2.65 (11 GHz)	[33]
Me-SSQ/CE	2.78–3.05	[13]
QF/SiR	3.35–3.42	[17]
PMEP-MHHPA	2.93.6 (10 MHz)	[3]
EHFHPSi	~2.96 (1 Hz)	[32]

**Table 2:** Composition and viscosity of BMSs.

Sample	Monomers		The Proportion of BCB Group (%) ^a^	Yield (%)	Viscosity (mPa s)
	BMDES/mmol	DMDES/mmol			
BMS-1	15	35	26.28	90.3	43.5
BMS-2	30	30	39.41	88.2	71.3
BMS-3	35	15	58.73	86.4	228.6
BMS-4	45	5	81.76	83.8	>2000

^a^ The proportion of BCB group was calculated from the integral data of ^1^H NMR spectra.

**Table 3:** The dielectric constant of BCB silicones with similar structure.

Resins	Dielectric Constant (1 MHz)	Ref.
CYC-BCB	2.77	[36]
LSOVBCB	2.27–2.48	[42]
AdaDBDVS	2.78	[37]
P(4-MB-co-1-MP)/DVS-BCB	2.52–2.65	[39]
R-Si-BCB	2.68 (5 GHz)	[43]
BPBVSi	2.76	[41]
BMS	2.77	This work

## Data Availability

All data from the study are included in the article.
